# Multivalued neutrosophic power partitioned Hamy mean operators and their application in MAGDM

**DOI:** 10.1371/journal.pone.0281734

**Published:** 2023-02-15

**Authors:** Muwen Wang, Tonghui Li, Yuan Tian, Kecheng Zhang

**Affiliations:** 1 School of Economics and Management, Shandong Agricultural University, Taian, China; 2 School of Business Administration, Shandong Women’s University, Jinan, China; Prince of Songkla University, THAILAND

## Abstract

The novel multivalued neutrosophic aggregation operators are proposed in this paper to handle the complicated decision-making situations with correlation between specific information and partitioned parameters at the same time, which are based on weighted power partitioned Hamy mean (WMNPPHAM) operators for multivalued neutrosophic sets (MNS) proposed by combining the Power Average and Hamy operators. Firstly, the power partitioned Hamy mean (PPHAM) is capable of capture the correlation between aggregation parameters and the relationship among attributes dividing several parts, where the attributes are dependent definitely within the interchangeable fragment, other attributes in divergent sections are irrelevant. Secondly, because MNS can effectively represent imprecise, insufficient, and uncertain information, we proposed the multivalued neutrosophic PMHAM (WMNPHAM) operator for MNS and its partitioned variant (WMNPPHAM) with the characteristics and examples. Finally, this multiple attribute group decision making (MAGDM) technique is proven to be feasible by comparing with the existing methods to confirm this method’s usefulness and validity.

## Introduction

The world is full of partial, imprecise, inconsistent, and uncertain data that can’t be characterized with precise numbers [1–3]. In order to deal with these complex problems, the MAGDM method can sort and adopt the superlative alternative from a set of complicated options [4]. Zadeh suggested fuzzy set to solve MAGDM problems in order to decrease information loss and increase assessment accuracy [5]. However, main limitation of fuzzy set is that it can’t handle complex fuzzy information adequately because its membership limit is only one value. As a result, Atanassov expanded fuzzy set to the intuitionistic fuzzy set [6–8]. However, in the face of conflicting, partial, and uncertain data, the foregoing has some limitations. Smarandache proposed the notion of neutrosophic sets to address this issue, which incorporated an independent indeterminacy membership function [9]. Such as the generalization of fuzzy sets, the single-valued neutrosophic set and multivalued neutrosophic sets are some of the achievements in this subject [10–17].

Because the decision makers typically cannot generate precise assessment values for every membership due to limited knowledge and experience [18–20], the MNS can manage the unclear information that has more than one value of membership offered by certain decision makers. As a result, MNS can cope with convoluted fuzzy information better than other fuzzy sets, but little research in this subject has been conducted, thus it is critical to investigate the MADM or MAGDM approach using MNS.

Aggregation operators, as a critical tool for solving MADM or MAGDM issues, may combine information from all qualities and decision makers and rate the options [21–29]. However, there has been little progress in the research of MNS aggregation operators thus far. As previously stated, ranking systems such as PROMETHEE [30, 31], TODIM [32, 33], and others were unable to produce complete values for many options. Furthermore, approaches based on aggregation operators can produce both ranking results and complete values [34].

Existing MAGDM approaches are unable to capture complex relationship patterns between characteristics properly. However, actual MADM or MAGDM situations with many attribute correlations need the use of developing operators as Power average operator, Maclaurin symmetric mean operator [35–39]. When compared with Heronian mean which was proposed by Hara [40], the Hamy mean operator can manage the interrelationships between numerous qualities more flexibly. Furthermore, Liu [41], Wu [42] point out that Hamy mean operator is a more powerful extension of MSM. However, the classic Hamy mean operator has several limitations, such as the fact that it can only aggregate crisp integers and cannot directly establish relationships among multiple-input arguments with partition structure.

It is critical to expand the Hamy mean operator to handle complicated MAGDM issues with ambiguous information and interrelationships of attributes, the novel multi-valued neutrosophic power partitioned hamy mean (MNPPHM) operator is presented in this paper, which is based on the Hamy operator. As a result, our research concentrates on the nominated operators to build the MAGDM technique using MNS. The contributions are:

1. The PPHAM operator is extended to improve the ability of existing operators to handle the specific information and partitioned parameters.
2. The PHAM operator (WMNPHAM) is presented with MNS, as well as its partitioned operator (WMNPPHAM).
3. The developed operators’ properties are demonstrated with numerous unique examples.
4. A practical MAGDM approach is generalized with the WMNPPHAM operator.
5. The applicability and efficacy of the provided MAGDM technique is demonstrated.

The following is the structure of this paper. We introduce the fundamental principles and operating norms of MNS and Hamy mean operator operators in Section 2. Section 3 proposes the WMNPHAM and WMNPPHAM. In Section 4, we construct MAGDM technique with these operators. Section 5 provides an example that compares the proposed method to existing ways to demonstrate its viability. Finally, the findings are described in Section 6.

## Preliminaries

### The neutrosophic fuzzy set

Definition 1. As a universe of objects K, where *q* is a generic element in K, the single valued neutrosophic set *Q* in *K* is defined as:

$$\text{Q}=\left\{q\left(U_{K}\left(q\right),D_{K}\left(q\right),M_{K}\left(q\right)\right)|q\in K\right\}$$ (1)

where U_K_(*q*), D_K_(*q*) and M_K_(*q*) denote the truth-membership, the indeterminacy-membership and the falsity-membership of the element *x* ∈ *X* to the set K respectively. For each point *q* in Q, we have U_K_(*q*), D_K_(*q*), M_K_(*q*) ∈ [0,1], 0 ≤ U_K_(*q*) + D_K_(*q*) + M_K_(*q*) ≤ 3.

For simplicity, we may utilize the simpler form *Q* = (*U*_*q*_, *D*_*q*_, *M*_*q*_) to represent single valued neutrosophic set, and the element q can be termed as a single valued neutrosophic number.

Definition 2. K is a nonempty fixed set, the multivalued neutrosophic set (MNS) in *Q* could be characterized as:

$$\text{I}=\left\{\langle \text{q,}\tilde{\text{u}}(q),\tilde{d}(q),\tilde{l}(q)\rangle ,q\in K\right\}$$ (2)

where $\tilde{u}\left(q\right)=\{\tilde{\mu }|\tilde{\mu }\in \tilde{u}\left(x\right)\}$, $\tilde{d}\left(q\right)=\{\tilde{d}|\tilde{\rho }\in \tilde{d}\left(x\right)\}$ and $\tilde{l}(q)=\{\tilde{\lambda }|\tilde{\lambda }\in \tilde{l}(q)\}$ are three sets with some values in interval [0,1], and satisfying the limits: $\tilde{\mu },\tilde{\rho },\tilde{\lambda }\in \left[0,1\right]$ and $0\leq \text{sup}{\tilde{\mu }}^{+}+\text{sup}{\tilde{\rho }}^{+}+\text{sup}{\tilde{\lambda }}^{+}\leq 3$.

The $\tilde{i}=\left\{\tilde{\mu }(q),\tilde{\rho }(q),\tilde{\lambda }(q)\right\}$ is known as a multivalued neutrosophic number which is indicated by the simplified symbol $\tilde{i}=\left\{\tilde{\mu },\tilde{\rho },\tilde{\lambda }\right\}$.

Definition 3. Let *I* ∈ *MNS*, the complement of an MNS may be characterized as *I*^*c*^ stated as follows:

$$I^{c}=\langle \cup_{\mu \in U_{I}}\left\{\lambda \right\},\cup_{\rho \in D_{I}}\left\{1-\rho \right\},\cup_{\lambda \in L_{I}}\left\{\mu \right\}\rangle$$ (3)

Definition 4. Two MNS ${\tilde{I}}_{1}=\left\{{\tilde{u}}_{1},{\tilde{d}}_{1},{\tilde{l}}_{1}\right\}$ and ${\tilde{I}}_{2}=\left\{{\tilde{u}}_{2},{\tilde{d}}_{2},{\tilde{l}}_{2}\right\}$, and *k* > 0, the basic operations can be defined as:

1.
$${\tilde{i}}_{1}⊕{\tilde{i}}_{2}=\left\{{\tilde{u}}_{1}⊕{\tilde{u}}_{2},{\tilde{d}}_{1}⊗{\tilde{d}}_{2},{\tilde{l}}_{1}⊗{\tilde{l}}_{2}\right\}=\underset{\begin{array}{l} {\tilde{\mu }}_{1}\in u_{1},{\tilde{\rho }}_{1}\in d_{1},{\tilde{\lambda }}_{1}\in l_{1},\\ {\tilde{\mu }}_{2}\in u_{2},{\tilde{\rho }}_{2}\in d_{2},{\tilde{\lambda }}_{2}\in l_{2}, \end{array}}{\cup }\left\{\mu_{1}+\mu_{2}-\mu_{1}\mu_{2},\rho_{1}\rho_{2},\lambda_{1}\lambda_{2}\right\};$$ (4)
2.
$${\tilde{i}}_{1}⊗{\tilde{i}}_{2}=\left\{{\tilde{u}}_{1}⊗{\tilde{u}}_{2},{\tilde{d}}_{1}⊕{\tilde{d}}_{2},{\tilde{l}}_{1}⊕{\tilde{l}}_{2}\right\}=\underset{\begin{array}{l} {\tilde{\mu }}_{1}\in u_{1},{\tilde{\rho }}_{1}\in d_{1},{\tilde{\lambda }}_{1}\in l_{1},\\ {\tilde{\mu }}_{2}\in u_{2},{\tilde{\rho }}_{2}\in d_{2},{\tilde{\lambda }}_{2}\in l_{2}, \end{array}}{\cup }\left\{\mu_{1}\mu_{2},\rho_{1}+\rho_{2}-\rho_{1}\rho_{2},\lambda_{1}+\lambda_{2}-\lambda_{1}\lambda_{2}\right\};$$ (5)
3.
$$k{\tilde{i}}_{1}=\underset{{\tilde{\mu }}_{1}\in u_{1},{\tilde{\rho }}_{1}\in d_{1},{\tilde{\lambda }}_{1}\in l_{1}}{\cup }\left\{1-{\left(1-\mu_{1}\right)}^{k},\rho_{1}^{k},\lambda_{1}^{k}\right\};$$ (6)
4.
$${\tilde{i}}_{1}^{k}=\underset{{\tilde{\mu }}_{1}\in u_{1},{\tilde{\rho }}_{1}\in d_{1},{\tilde{\lambda }}_{1}\in l_{1}}{\cup }\left\{\mu_{1}^{k},1-{\left(1-\upsilon_{1}\right)}^{k},1-{\left(1-\lambda_{1}\right)}^{k}\right\}$$ (7)

Definition 5. Such any MNS $\tilde{i}$,

$$G\left({\tilde{i}}_{i}\right)=\frac{1}{l_{{\tilde{u}}_{i}}\cdot l_{{\tilde{d}}_{i}}\cdot l_{{\tilde{l}}_{i}}}\sum_{\gamma_{i}\in {\tilde{\text{u}}}_{i},\mu_{i}\in {\tilde{\text{d}}}_{i},\lambda_{i}\in {\tilde{l}}_{i},}\left(\mu_{i}+\left(1-\rho_{i}\right)+\left(1-\lambda_{i}\right)\right)/3$$ (8)

is regarded as the score function of ñ, where $l_{{\tilde{u}}_{A}}$,$l_{{\tilde{d}}_{A}}$ and $\;l_{{\tilde{l}}_{A}}$ reflects the numbers of the values in ${\tilde{u}}_{i}$, ${\tilde{d}}_{i}$, ${\tilde{l}}_{i}$.

Definition 6. For any a MNS $\tilde{i}$,

$$B(\tilde{i})=\frac{1}{l_{{\tilde{u}}_{i}}\cdot l_{{\tilde{d}}_{i}}\cdot l_{{\tilde{l}}_{i}}}\sum_{\mu_{i}\in {\tilde{u}}_{i},\rho_{i}\in {\tilde{d}}_{i},\lambda_{i}\in {\tilde{l}}_{i},}\left(\mu_{i}+\rho_{i}+\lambda_{i}\right)/3$$ (9)

Assume ${\tilde{i}}_{1}=\left\{{\tilde{u}}_{1},{\tilde{d}}_{1},{\tilde{l}}_{1}\right\}$ and ${\tilde{i}}_{2}=\left\{{\tilde{u}}_{2},{\tilde{d}}_{2},{\tilde{l}}_{2}\right\}$ are two MNS, the comparison algorithm of MNS is determined by the following equations:

1. If $G({\tilde{i}}_{1})\geq G({\tilde{i}}_{2})$ and $B\left({\tilde{i}}_{1}\right)>B\left({\tilde{i}}_{2}\right)$, then ${\tilde{i}}_{1}≻{\tilde{i}}_{2}$
2. If $G({\tilde{i}}_{1})=G({\tilde{i}}_{2})$ and $B\left({\tilde{i}}_{1}\right)=B\left({\tilde{i}}_{2}\right)$, then ${\tilde{i}}_{1}\approx {\tilde{i}}_{2}$
3. If $G({\tilde{i}}_{1})\leq G({\tilde{i}}_{2})$ and $B\left({\tilde{i}}_{1}\right)<B\left({\tilde{i}}_{2}\right)$, then ${\tilde{i}}_{1}≺{\tilde{i}}_{2}$.

Definition 7. Assume $P=\left\{{\tilde{U}}_{P},{\tilde{D}}_{P},{\tilde{l}}_{P}\right\}\;\text{and}\;Q=\left\{{\tilde{U}}_{Q},{\tilde{D}}_{Q},{\tilde{L}}_{Q}\right\}$, then the Hamming distance between them can be expressed in terms:

$$\begin{array}{l} d(P,Q)=\frac{1}{6}(\underset{\mu_{P}\in {\tilde{U}}_{p}}{\text{max}}\underset{\mu_{Q}\in {\tilde{U}}_{Q}}{\text{min}}|\mu_{P}-\mu_{Q}|+\underset{\mu_{Q}\in {\tilde{U}}_{Q}}{\text{max}}\underset{\mu_{P}\in {\tilde{U}}_{P}}{\text{min}}|\mu_{Q}-\mu_{A}|\\ +\underset{\rho_{P}\in {\tilde{D}}_{P}}{\text{max}}\underset{\rho_{Q}\in {\tilde{D}}_{Q}}{\text{min}}|\rho_{P}-\rho_{Q}|+\underset{\rho_{P}\in {\tilde{D}}_{P}}{\text{max}}\underset{\rho_{Q}\in {\tilde{D}}_{Q}}{\text{min}}|\rho_{Q}-\rho_{P}|\\ +\underset{\lambda_{A}\in {\tilde{L}}_{A}}{\text{max}}\underset{\lambda_{B}\in {\tilde{L}}_{B}}{\text{min}}|\lambda_{P}-\lambda_{Q}|+\underset{\lambda_{B}\in {\tilde{L}}_{B}}{\text{max}}\underset{\lambda_{Q}\in {\tilde{L}}_{Q}}{\text{min}}|\lambda_{Q}-\lambda_{P}|) \end{array}$$ (10)

### Hamy mean operator

Definition 8. The form of Hamy mean operator is:

$$HAM^{\left(j\right)}\left(w_{1},w_{2},\ldots ,w_{n}\right)=\frac{\sum_{1\leq i_{1},\ldots ,i_{j}\leq n}{\left(\prod_{m=1}^{j}w_{i_{m}}\right)}^{1/j}}{C_{n}^{j}}$$ (11)

Where (*i*_1_, *i*_2_, …,*i*_*j*_) explores every j-tuple combination and $C_{n}^{j}$ is the binomial coefficient, and $C_{n}^{j}=\frac{n!}{j!\left(n-j\right)!}$.

Obviously, the Hamy mean operator has several characteristics:

1. *HAM*^(*j*)^ (0,0,…,0) = 0, *HAM*^(*j*)^(*w*, *w*,…,*w*) = *w*;
2. *HAM*^(*j*)^ (*w*_1_, *w*_2_,…,*w*_*n*_) ≤ *HAM*^(*j*)^ (*y*_1_, *y*_2_,…,*y*_*n*_), *if w*_*i*_ ≤ *y*_*i*_ *for all i*;
3. min{*w*_*i*_} ≤ *HAM*^(*j*)^ (*w*_1_, *w*_2_,…,*w*_*n*_) ≤ max{*w*_*i*_}.

### Power aggregation operators

Definition 9. The Power aggregation operator is the mapping *R*^*n*^ → *R* as:

$$PA\left(\alpha_{1},\alpha_{2},\ldots ,\alpha_{n}\right)=\frac{\sum_{i=1}^{n}\left(1+S\left(\alpha_{i}\right)\right)\alpha_{i}}{\sum_{i=1}^{n}\left(1+S\left(\alpha_{i}\right)\right)}$$ (12)

Where $S\left(\alpha_{i}\right)=\sum_{i=1,j\neq i}^{n}Sup\left(\alpha_{i,}\alpha_{j}\right)$, and *Sup*(*α*_*i*_, *α*_*j*_) is the support as *α*_*i*_ from *α*_*j*_. And certain qualities are detailed as follows:

1. *Sup*(*α*_*i*_, *α*_*j*_) ∈ [0,1];
2. *Sup*(*α*_*i*_, *α*_*j*_) = *Sup*(*α*_*j*_, *α*_*i*_)
3. *Sup*(*α*_*i*_, *α*_*j*_) ≥ *Sup*(*α*_*e*_, *α*_*f*_), *if* |*α*_*e*_ − *α*_*f*_|<|*α*_*i*_ − *α*_*j*_|.

### Partitioned Hamy mean (PHAM) operator

Definition 10. The Partitioned Hamy mean operator (PHAM) is expressed in the form as:

$$PHAM^{\left(p\right)}\left(x_{1},x_{2},\ldots ,x_{n}\right)=\frac{1}{l}\sum_{r=1}^{l}\left(\frac{\sum_{1\leq i_{1},\ldots ,i_{p}\leq q}{\left(\prod_{j=1}^{p}x_{i_{j}}\right)}^{1/p}}{C_{q}^{p}}\right)$$ (13)

Where (*i*_1_, *i*_2_, …,*i*_*p*_) explores the whole p-tuple combination and $C_{q}^{p}$ is the binomial coefficient, and $C_{q}^{p}=\frac{q!}{p!\left(q-p\right)!}$.

## Hamy mean operators based on multivalued neutrosophic sets

In summary, we will investigate the Hamy mean operator and Power aggregation operator to deal with MNS and build MNPPHAM operator and WMNPPHAM operator, as well as explain various attributes and specific circumstances of these new operators, with the operating regulations of MNS.

### MNPHAM operator

Definition 11. ${\tilde{n}}_{i}=\left\{{\tilde{u}}_{i},{\tilde{d}}_{i},{\tilde{l}}_{i}\right\}\left(i=1,2,\ldots ,n\right)$ are MNS, and *x* = 1,2,…,*m* The MNPHAM operator is described this way:

$$MNPHAM^{\left(x\right)}\left({\tilde{n}}_{1},{\tilde{n}}_{2},\cdots ,{\tilde{n}}_{n}\right)=\frac{1}{C_{n}^{x}}\left({\underset{}{\sum_{1\leq i_{1}<\cdots <i_{x}\leq n}\left(\prod {}_{j=1}^{x}\frac{n(1+T({\tilde{n}}_{i_{j}})){\tilde{n}}_{i_{j}}}{\sum {}_{z=1}^{n}\left(1+T\left({\tilde{n}}_{z}\right)\right)}\right)}}^{\frac{1}{x}}\right),$$ (14)

where $T\left({\tilde{n}}_{j}\right)=\sum {}_{\begin{array}{l} z=1\\ z\neq j \end{array}}^{n}Sup\left({\tilde{n}}_{z},{\tilde{n}}_{j}\right)$ is support degree, which meets:

1.
$Sup\left({\tilde{\text{n}}}_{z},{\tilde{\text{n}}}_{j}\right)\in \left[0,1\right];$

2.
$Sup\left({\tilde{\text{n}}}_{z},{\tilde{\text{n}}}_{j}\right)=Sup\left({\tilde{\text{n}}}_{j},{\tilde{\text{n}}}_{n}\right);$

3. $\text{if}\;\text{d}\left({\tilde{\text{n}}}_{z},{\tilde{\text{n}}}_{j}\right)\leq \text{d}\left({\tilde{\text{n}}}_{d},{\tilde{\text{n}}}_{y}\right)$, then $Sup\left({\tilde{\text{n}}}_{z},{\tilde{\text{n}}}_{j}\right)\geq Sup\left({\tilde{\text{n}}}_{d},{\tilde{\text{n}}}_{y}\right)$, where $\text{d}\left({\tilde{\text{n}}}_{z},{\tilde{\text{n}}}_{j}\right)$ indicate distance among any two neutrosophic sets signed by the Definition 7.

The denominator $C_{n}^{x}$ represents the binomial coefficient $\frac{n!}{x!\left(n-x\right)!}$ and *n* is the balancing coefficient in the preceding Eq (14), we could note

$$\sigma_{z}=\frac{\left(1+T({\tilde{n}}_{i_{j}})\right)}{\sum {}_{z=1}^{n}\left(1+T\left({\tilde{\text{n}}}_{z}\right)\right)}$$ (15)

then power weight vector is identified by (σ_1_, σ_2_,…,σ_*n*_). As a result, Eq (14) can be documented in the following simplified form:

$$MNPHAM^{\left(x\right)}\left({\tilde{n}}_{1},{\tilde{n}}_{2},\cdots ,{\tilde{n}}_{n}\right)=\frac{1}{C_{n}^{x}}\left({\underset{}{\sum_{1\leq i_{1}<\cdots <i_{x}\leq \text{n}}\left(\prod {}_{j=1}^{x}n\sigma_{i_{j}}{\tilde{\text{n}}}_{i_{j}}\right)}}^{\frac{1}{x}}\right)$$ (16)

The following theorems could be derived from the operational rules of the MNS:

Theorem 1. Let ${\tilde{n}}_{i}=\left\{{\tilde{u}}_{i},{\tilde{d}}_{i},{\tilde{l}}_{i}\right\}\left(i=1,2,\ldots ,n\right)$ be a MNS, the result of aggregation is still MNS.


MNPHAMxn˜1,n˜2,⋯,n˜n=1Cnx∑1≤i1<⋯<ix≤n∏j=1xnσijn˜ij1x=1−∏1≤i1<⋯<ix≤n1−∏j=1x∪γ˜ij∈t˜ij1−1−γijnσij1x1Cnx,∏1≤i1<⋯<ix≤n1−∏j=1x∪δ˜ij∈i˜ij1−δijnσij1x1Cnx,∏1≤i1<⋯<ix≤n1−∏j=1x∪η˜ij∈f˜ij1−ηijnσij1x1Cnx (17)

Proof. Since

1.
${\tilde{i}}_{1}⊕{\tilde{i}}_{2}=\left\{{\tilde{u}}_{1}⊕{\tilde{u}}_{2},{\tilde{d}}_{1}⊗{\tilde{d}}_{2},{\tilde{l}}_{1}⊗{\tilde{l}}_{2}\right\}=\underset{\begin{array}{l} {\tilde{\mu }}_{1}\in u_{1},{\tilde{\rho }}_{1}\in d_{1},{\tilde{\lambda }}_{1}\in l_{1},\\ {\tilde{\mu }}_{2}\in u_{2},{\tilde{\rho }}_{2}\in d_{2},{\tilde{\lambda }}_{2}\in l_{2}, \end{array}}{\cup }\left\{\mu_{1}+\mu_{2}-\mu_{1}\mu_{2},\rho_{1}\rho_{2},\lambda_{1}\lambda_{2}\right\};$

2.
${\tilde{i}}_{1}⊗{\tilde{i}}_{2}=\left\{{\tilde{u}}_{1}⊗{\tilde{u}}_{2},{\tilde{d}}_{1}⊕{\tilde{d}}_{2},{\tilde{l}}_{1}⊕{\tilde{l}}_{2}\right\}=\underset{\begin{array}{l} {\tilde{\mu }}_{1}\in u_{1},{\tilde{\rho }}_{1}\in d_{1},{\tilde{\lambda }}_{1}\in l_{1},\\ {\tilde{\mu }}_{2}\in u_{2},{\tilde{\rho }}_{2}\in d_{2},{\tilde{\lambda }}_{2}\in l_{2}, \end{array}}{\cup }\left\{\mu_{1}\mu_{2},\rho_{1}+\rho_{2}-\rho_{1}\rho_{2},\lambda_{1}+\lambda_{2}-\lambda_{1}\lambda_{2}\right\};$

3.
$k{\tilde{i}}_{1}=\underset{{\tilde{\mu }}_{1}\in u_{1},{\tilde{\rho }}_{1}\in d_{1},{\tilde{\lambda }}_{1}\in l_{1}}{\cup }\left\{1-{\left(1-\mu_{1}\right)}^{k},\rho_{1}^{k},\lambda_{1}^{k}\right\};$

4.
${\tilde{i}}_{1}^{k}=\underset{{\tilde{\mu }}_{1}\in u_{1},{\tilde{\rho }}_{1}\in d_{1},{\tilde{\lambda }}_{1}\in l_{1}}{\cup }\left\{\mu_{1}^{k},1-{\left(1-\rho_{1}\right)}^{k},1-{\left(1-\lambda_{1}\right)}^{k}\right\}$

we have

$$n\sigma_{i_{j}}{\tilde{n}}_{i_{j}}=\left(\cup_{{\tilde{\mu }}_{i_{j}}\in {\tilde{u}}_{i_{j}}}1-{\left(1-\mu_{i_{j}}\right)}^{n\sigma_{i_{j}}},\cup_{{\tilde{\rho }}_{i_{j}}\in {\tilde{d}}_{i_{j}}}\rho_{i_{j}}^{n\sigma_{i_{j}}},\cup_{{\tilde{\lambda }}_{i_{j}}\in {\tilde{l}}_{i_{j}}}\lambda_{i_{j}}^{n\sigma_{i_{j}}}\right);$$

and

$$\prod {}_{j=1}^{x}n\sigma_{i_{j}}{\tilde{\text{n}}}_{i_{j}}=\left(\prod_{j=1}^{x}\cup_{{\tilde{\mu }}_{i_{j}}\in {\tilde{u}}_{i_{j}}}\left(1-{\left(1-\mu_{i_{j}}\right)}^{n\sigma_{i_{j}}}\right),1-\left(\prod_{j=1}^{x}\cup_{{\tilde{\rho }}_{i_{j}}\in {\tilde{\rho }}_{i_{j}}}\left(1-{\left(\rho_{i_{j}}\right)}^{n\sigma_{i_{j}}}\right)\right),1-\left(\prod_{j=1}^{x}\cup_{{\tilde{\lambda }}_{i_{j}}\in {\tilde{l}}_{i_{j}}}\left(1-{\left(l_{i_{j}}\right)}^{n\sigma_{i_{j}}}\right)\right)\right)$$

so

$${\left(\prod {}_{j=1}^{x}n\sigma_{i_{j}}{\tilde{\text{n}}}_{i_{j}}\right)}^{\frac{1}{x}}=\left({\left(\prod_{j=1}^{x}\cup_{{\tilde{\mu }}_{i_{j}}\in {\tilde{u}}_{i_{j}}}\left(1-{\left(1-\mu_{i_{j}}\right)}^{n\sigma_{i_{j}}}\right)\right)}^{\frac{1}{x}},1-{\left(\prod_{j=1}^{x}\cup_{{\tilde{\rho }}_{i_{j}}\in {\tilde{d}}_{i_{j}}}\left(1-{\left(\rho_{i_{j}}\right)}^{n\sigma_{i_{j}}}\right)\right)}^{\frac{1}{x}},1-{\left(\prod_{j=1}^{x}\cup_{{\tilde{\lambda }}_{i_{j}}\in {\tilde{l}}_{i_{j}}}\left(1-{\left(\lambda_{i_{j}}\right)}^{n\sigma_{i_{j}}}\right)\right)}^{\frac{1}{x}}\right)$$

then

1Cnx∑1≤i1<⋯<ix≤n∏j=1xnσijn˜ij1x=1−∏1≤i1<⋯<ix≤n1−∏j=1x∪μ˜ij∈u˜ij1−1−μijnσij1x1Cnx,∏1≤i1<⋯<ix≤n1−∏j=1x∪ρ˜ij∈d˜ij1−ρijnσij1x1Cnx,∏1≤i1<⋯<ix≤n1−∏j=1x∪λ˜ij∈l˜ij1−λijnσij1x1Cnx

Therefore,

MNPHAMxn˜1,n˜2,⋯,n˜n=1Cnx∑1≤i1<⋯<ix≤n∏j=1xnσijn˜ij1x=1−∏1≤i1<⋯<ix≤n1−∏j=1x∪γ˜ij∈t˜ij1−1−γijnσij1x1Cnx,∏1≤i1<⋯<ix≤n1−∏j=1x∪δ˜ij∈i˜ij1−δijnσij1x1Cnx,∏1≤i1<⋯<ix≤n1−∏j=1x∪η˜ij∈f˜ij1−ηijnσij1x1Cnx

In addition, the NPHAM has some features as:

(1) Theorem 2 (Idempotency). ${\tilde{n}}_{i}=\tilde{n}=\left\{\tilde{u},\tilde{d},\tilde{l}\right\}$, for (*i* = 1, 2,…,*n*), then

$$MNPHAM^{\left(x\right)}\left({\tilde{n}}_{1},{\tilde{n}}_{2},\cdots ,{\tilde{n}}_{n}\right)=\tilde{n}=\left\{\tilde{u},\tilde{d},\tilde{l}\right\}.$$ (18)

Proof. Since ${\tilde{n}}_{i}=\tilde{n}=\left\{\tilde{u},\tilde{d},\tilde{l}\right\}$, for (*i* = 1, 2,…,*n*), then

$$n\sigma_{i_{j}}=\frac{n(1+T({\tilde{\text{n}}}_{i_{j}}))}{\sum {}_{z=1}^{n}(1+T\left({\tilde{\text{n}}}_{z}\right))}=1$$

So, according to Theorem 1, we have

MNPHAMxn˜1,n˜2,⋯,n˜n=1Cnx∑1≤i1<⋯<ix≤n∏j=1xnσijn˜ij1x=1−∏1≤i1<⋯<ix≤n1−∏j=1x∪μ˜ij∈u˜ij1−1−μijnσij1x1Cnx,∏1≤i1<⋯<ix≤n1−∏j=1x∪ρ˜ij∈d˜ij1−ρijnσij1x1Cnx,∏1≤i1<⋯<ix≤n1−∏j=1x∪λ˜ij∈l˜ij1−λijnσij1x1Cnx

=∪μ˜ij∈u˜ij,ρ˜ij∈d˜ij,λ˜ij∈l˜ij1−1−1−1−μijnσij1x1Cnx,1−1−ρijnσij1x1Cnx,1−1−λijnσij1x1Cnx

$$=\cup_{{\tilde{\mu }}_{i_{j}}\in {\tilde{u}}_{i_{j}},{\tilde{\rho }}_{i_{j}}\in {\tilde{d}}_{i_{j}},{\tilde{\lambda }}_{i_{j}}\in {\tilde{l}}_{i_{j}}}\left(\mu ,\rho ,\lambda \right)=\tilde{n}=\left\{\tilde{u},\tilde{d},\tilde{l}\right\}$$

(2) Theorem 3 (monotonicity). Let ${ñ}_{i}(i=\;1,2,\cdots ,n)$ and ${ñ}^{\prime }{}_{i}(i=1,2,\cdots ,n)$ be two MNS, and suppose $\gamma_{i}\geq {\gamma^{\prime }}_{i},\delta_{i}\leq {\delta^{\prime }}_{i}$ and $\eta_{i}\leq {\eta^{\prime }}_{i}$ for all *i*, then

$$MNPHAM^{\left(x\right)}\left({\tilde{n}}_{1},{\tilde{n}}_{2},\cdots ,{\tilde{n}}_{n}\right)\geq MNPHAM^{\left(x\right)}\left({\tilde{n}}^{\prime }{}_{1},{\tilde{n}}^{\prime }{}_{2},\cdots ,{\tilde{n}}^{\prime }{}_{n}\right).$$ (19)

Proof. (1)Since $\mu_{i}\geq {\mu^{\prime }}_{i}$ for all *i*, then

$$\mu_{i_{j}}\geq {\mu^{\prime }}_{i_{j}},1-\mu_{i_{j}}\leq 1-{\mu^{\prime }}_{i_{j}}$$

$${\left(\prod_{j=1}^{x}\cup_{{\tilde{\mu }}_{i_{j}}\in {\tilde{u}}_{i_{j}}}\left(1-{\left(1-\mu_{i_{j}}\right)}^{n\sigma_{i_{j}}}\right)\right)}^{\frac{1}{x}}\geq {\left(\prod_{j=1}^{x}\cup_{{\tilde{\mu }}_{i_{j}}\in {\tilde{u}}_{i_{j}}}\left(1-{\left(1-{\mu^{\prime }}_{i_{j}}\right)}^{n\sigma_{i_{j}}}\right)\right)}^{\frac{1}{x}}$$

$$1-{\left(\prod_{j=1}^{x}\cup_{{\tilde{\mu }}_{i_{j}}\in {\tilde{u}}_{i_{j}}}\left(1-{\left(1-\mu_{i_{j}}\right)}^{n\sigma_{i_{j}}}\right)\right)}^{\frac{1}{x}}\leq 1-{\left(\prod_{j=1}^{x}\cup_{{\tilde{\mu }}_{i_{j}}\in {\tilde{u}}_{i_{j}}}\left(1-{\left(1-{\mu^{\prime }}_{i_{j}}\right)}^{n\sigma_{i_{j}}}\right)\right)}^{\frac{1}{x}}$$

$$\prod_{1\leq i_{1}<\cdots <i_{x}\leq \text{n}}^{}\left(1-{\left(\prod_{j=1}^{x}\cup_{{\tilde{\mu }}_{i_{j}}\in {\tilde{u}}_{i_{j}}}\left(1-{\left(1-\mu_{i_{j}}\right)}^{n\sigma_{i_{j}}}\right)\right)}^{\frac{1}{x}}\right)\leq \prod_{1\leq i_{1}<\cdots <i_{x}\leq \text{n}}^{}\left(1-{\left(\prod_{j=1}^{x}\cup_{{\tilde{\mu }}_{i_{j}}\in {\tilde{u}}_{i_{j}}}\left(1-{\left(1-{\mu^{\prime }}_{i_{j}}\right)}^{n\sigma_{i_{j}}}\right)\right)}^{\frac{1}{x}}\right)$$

∏1≤i1<⋯<ix≤n1−∏j=1x∪μ˜ij∈u˜ij1−1−μijnσij1x1Cnx≤∏1≤i1<⋯<ix≤n1−∏j=1x∪μ˜ij∈u˜ij1−1−μ′ijnσij1x1Cnx

So

1−∏1≤i1<⋯<ix≤n1−∏j=1x∪μ˜ij∈u˜ij1−1−μijnσij1x1Cnx≥1−∏1≤i1<⋯<ix≤n1−∏j=1x∪μ˜ij∈u˜ij1−1−μ′ijnσij1x1Cnx

(2) Since $\rho_{i}\leq {\rho^{\prime }}_{i}$ for all *i*, then

$$\rho_{i_{j}}\leq {\rho^{\prime }}_{i_{j}},1-\rho_{i_{j}}\geq 1-{\rho^{\prime }}_{i_{j}}$$

$${\left(\prod_{j=1}^{x}\cup_{{\tilde{\rho }}_{i_{j}}\in {\tilde{d}}_{i_{j}}}\left(1-{\left(\rho_{i_{j}}\right)}^{n\sigma_{i_{j}}}\right)\right)}^{\frac{1}{x}}\geq {\left(\prod_{j=1}^{x}\cup_{{\tilde{\rho }}_{i_{j}}\in {\tilde{d}}_{i_{j}}}\left(1-{\left({\rho^{\prime }}_{i_{j}}\right)}^{n\sigma_{i_{j}}}\right)\right)}^{\frac{1}{x}}$$

$$\prod_{1\leq i_{1}<\cdots <i_{x}\leq \text{n}}\left(1-{\left(\prod_{j=1}^{x}\cup_{{\tilde{\rho }}_{i_{j}}\in {\tilde{d}}_{i_{j}}}\left(1-{\left(\rho_{i_{j}}\right)}^{n\sigma_{i_{j}}}\right)\right)}^{\frac{1}{x}}\right)\leq \prod_{1\leq i_{1}<\cdots <i_{x}\leq \text{n}}\left(1-{\left(\prod_{j=1}^{x}\cup_{{\tilde{\rho }}_{i_{j}}\in {\tilde{d}}_{i_{j}}}\left(1-{\left({\rho^{\prime }}_{i_{j}}\right)}^{n\sigma_{i_{j}}}\right)\right)}^{\frac{1}{x}}\right)$$

So

$${\left(\prod_{1\leq i_{1}<\cdots <i_{x}\leq \text{n}}\left(1-{\left(\prod_{j=1}^{x}\cup_{{\tilde{\rho }}_{i_{j}}\in {\tilde{d}}_{i_{j}}}\left(1-{\left(\rho_{i_{j}}\right)}^{n\sigma_{i_{j}}}\right)\right)}^{\frac{1}{x}}\right)\right)}^{\frac{1}{C_{n}^{x}}}\leq {\left(\prod_{1\leq i_{1}<\cdots <i_{x}\leq \text{n}}\left(1-{\left(\prod_{j=1}^{x}\cup_{{\tilde{\rho }}_{i_{j}}\in {\tilde{d}}_{i_{j}}}\left(1-{\left({\rho^{\prime }}_{i_{j}}\right)}^{n\sigma_{i_{j}}}\right)\right)}^{\frac{1}{x}}\right)\right)}^{\frac{1}{C_{n}^{x}}}$$

(3) Since $\lambda_{i}\leq {\lambda^{\prime }}_{i}$, for all *i*, then

$$\lambda_{i_{j}}\leq \lambda_{i_{j}}^{\prime },1-\lambda_{i_{j}}\geq 1-\lambda_{i_{j}}^{\prime }$$

$${\left(\prod_{j=1}^{x}\cup_{{\tilde{\lambda }}_{i_{j}}\in {\tilde{l}}_{i_{j}}}\left(1-{\left(\lambda_{i_{j}}\right)}^{n\sigma_{i_{j}}}\right)\right)}^{\frac{1}{x}}\geq {\left(\prod_{j=1}^{x}\cup_{{\tilde{\lambda }}_{i_{j}}\in {\tilde{l}}_{i_{j}}}\left(1-{\left(\lambda_{i_{j}}\right)}^{n\sigma_{i_{j}}}\right)\right)}^{\frac{1}{x}}$$

$$\prod_{1\leq i_{1}<\cdots <i_{x}\leq \text{n}}\left(1-{\left(\prod_{j=1}^{x}\cup_{{\tilde{\lambda }}_{i_{j}}\in {\tilde{l}}_{i_{j}}}\left(1-{\left(\lambda_{i_{j}}\right)}^{n\sigma_{i_{j}}}\right)\right)}^{\frac{1}{x}}\right)\leq \prod_{1\leq i_{1}<\cdots <i_{x}\leq \text{n}}\left(1-{\left(\prod_{j=1}^{x}\cup_{{\tilde{\lambda }}_{i_{j}}\in {\tilde{l}}_{i_{j}}}\left(1-{\left({\lambda^{\prime }}_{i_{j}}\right)}^{n\sigma_{i_{j}}}\right)\right)}^{\frac{1}{x}}\right)$$

So

$${\left(\prod_{1\leq i_{1}<\cdots <i_{x}\leq \text{n}}\left(1-{\left(\prod_{j=1}^{x}\cup_{{\tilde{\lambda }}_{i_{j}}\in {\tilde{l}}_{i_{j}}}\left(1-{\left(\lambda_{i_{j}}\right)}^{n\sigma_{i_{j}}}\right)\right)}^{\frac{1}{x}}\right)\right)}^{\frac{1}{C_{n}^{x}}}\leq {\left(\prod_{1\leq i_{1}<\cdots <i_{x}\leq \text{n}}\left(1-{\left(\prod_{j=1}^{x}\cup_{{\tilde{\lambda }}_{i_{j}}\in {\tilde{l}}_{i_{j}}}\left(1-{\left({\lambda^{\prime }}_{i_{j}}\right)}^{n\sigma_{i_{j}}}\right)\right)}^{\frac{1}{x}}\right)\right)}^{\frac{1}{C_{n}^{x}}}$$

we can get

$$MNPHAM^{\left(x\right)}\left({\tilde{n}}_{1},{\tilde{n}}_{2},\cdots ,{\tilde{n}}_{n}\right)\geq MNPHAM^{\left(x\right)}\left({\tilde{n}}^{\prime }{}_{1},{\tilde{n}}^{\prime }{}_{2},\cdots ,{\tilde{n}}^{\prime }{}_{n}\right).$$

(3) Theorem 4 (Boundedness). The MNHPHM operator situates within:

$$min\left({\tilde{n}}_{1},{\tilde{n}}_{2},\cdots ,{\tilde{n}}_{n}\right)\leq MNHFHM\left({\tilde{n}}_{1},{\tilde{n}}_{2},\cdots ,{\tilde{n}}_{n}\right)\leq max\left({\tilde{n}}_{1},{\tilde{n}}_{2},\cdots ,{\tilde{n}}_{n}\right)$$ (20)

Proof. Let $m=min\left({\tilde{n}}_{1},{\tilde{n}}_{2},\cdots ,{\tilde{n}}_{n}\right),M=max\left({\tilde{n}}_{1},{\tilde{n}}_{2},\cdots ,{\tilde{n}}_{n}\right)$, since $m\leq {\tilde{n}}_{i_{j}}\leq M$,

$$m=min\left({\tilde{n}}_{1},{\tilde{n}}_{2},\cdots ,{\tilde{n}}_{n}\right)\leq MNPHAM\left({\tilde{n}}_{1},{\tilde{n}}_{2},\cdots ,{\tilde{n}}_{n}\right)$$

and

$$MNPHAM\left({\tilde{n}}_{1},{\tilde{n}}_{2},\cdots ,{\tilde{n}}_{n}\right)\leq max\left({\tilde{n}}_{1},{\tilde{n}}_{2},\cdots ,{\tilde{n}}_{n}\right)=M$$

then

$$m=min\left({\tilde{n}}_{1},{\tilde{n}}_{2},\cdots ,{\tilde{n}}_{n}\right)$$

$$\leq MNPHAM\left({\tilde{n}}_{1},{\tilde{n}}_{2},\cdots ,{\tilde{n}}_{n}\right)$$

$$\leq max\left({\tilde{n}}_{1},{\tilde{n}}_{2},\cdots ,{\tilde{n}}_{n}\right)$$

### WMNPHAM operator

Definition 12.${\tilde{n}}_{i}=\left\{{\tilde{u}}_{i},{\tilde{d}}_{i},{\tilde{l}}_{i}\right\}$ is a MNS, and weighted neutrosophic set power Hamy mean operator (WMNPHAM) is expressed in the form.

$$\begin{array}{l} WMNPHAM^{\left(x\right)}\left({\tilde{n}}_{1},{\tilde{n}}_{2},\cdots ,{\tilde{n}}_{n}\right)\\ =\frac{1}{C_{n}^{x}}\left({\underset{}{\sum_{1\leq i_{1}<\cdots <i_{x}\leq n}\left(\prod {}_{j=1}^{x}n\tau_{i_{j}}{\tilde{\text{n}}}_{i_{j}}\right)}}^{\frac{1}{x}}\right) \end{array}$$ (21)

where $\tau_{i}=\frac{\omega_{i}\left(1+T\left({\tilde{\text{n}}}_{i}\right)\right)}{\sum {}_{z=1}^{m}\omega_{i}\left(1+T\left({\tilde{\text{n}}}_{z}\right)\right)}$, $T\left({\tilde{\text{n}}}_{j}\right)=\sum {}_{\begin{array}{l} z=1\\ z\neq j \end{array}}^{m}Sup\left({\tilde{\text{n}}}_{z},{\tilde{\text{n}}}_{j}\right)$ which meet the specified criteria:

1.
$Sup\left({\tilde{n}}_{z},{\tilde{n}}_{j}\right)\in \left[0,1\right];$

2.
$Sup\left({\tilde{n}}_{z}\text{,}{\tilde{n}}_{j}\right)=Sup\left({\tilde{n}}_{j},{\tilde{n}}_{n}\right);$

3. $\text{if}\;\text{d}\left({\tilde{\text{n}}}_{z},{\tilde{\text{n}}}_{j}\right)\leq \text{d}\left({\tilde{\text{n}}}_{d},{\tilde{\text{n}}}_{y}\right)$ then $Sup\left({\tilde{\text{n}}}_{z},{\tilde{\text{n}}}_{j}\right)\geq Sup\left({\tilde{\text{n}}}_{d},{\tilde{\text{n}}}_{y}\right)$, where $d\left({\tilde{n}}_{z},{\tilde{n}}_{j}\right)$ represent the measure of distance in definition 7. *ω* = (*ω*_1_,*ω*_2_,…,*ω*_*n*_)^T^ is the weight vector of ${\tilde{n}}_{i}\left(i_{1},i_{2},\ldots ,i_{n}\right)$ such that *ω*_*i*_ ∈ [0,1] and $\sum {}_{i=1}^{n}\omega_{i}=1\left(i_{1},i_{2},\ldots ,i_{n}\right)$.

Theorem 5. Assume ${\tilde{n}}_{i}=\left\{{\tilde{u}}_{i},{\tilde{d}}_{i},{\tilde{l}}_{i}\right\}$ be MNS, So the result of aggregation is still MNS.


WMNPHAMxn˜1,n˜2,⋯,n˜n=1−∏1≤i1<⋯<ix≤n1−∏j=1x∪μij∈uij1−1−μijnτij1x1Cnx,∏1≤i1<⋯<ix≤n1−∏j=1x∪ρij∈dij1−ρijnτij1x1Cnx,∏1≤i1<⋯<ix≤n1−∏j=1x∪λij∈lij1−λijnτij1x1Cnx. (22)

Proof. This theorem’s proof is analogous to that of Theorem 1.

(1)Theorem 6 (Idempotency).${\tilde{n}}_{i}=\tilde{n}=\left\{\tilde{u},\tilde{d},\tilde{l}\right\}$, for all i, we have

$$WNPHAM^{\left(x\right)}\left({\tilde{n}}_{1},{\tilde{n}}_{2},\cdots ,{\tilde{n}}_{n}\right)=\tilde{n}=\left\{\tilde{u},\tilde{d},\tilde{l}\right\}$$ (23)

(2) Theorem 7 (monotonicity). If ${\tilde{n}}_{i}\left(i=1,2,\cdots ,n\right)$ and ${\tilde{n}}^{\prime }{}_{i}\left(i=1,2,\cdots ,n\right)$ be two neutrosophic sets, and suppose $\mu_{i}\geq {\mu^{\prime }}_{i},\rho_{i}\leq {\rho^{\prime }}_{i}$ and $\lambda_{i}\leq {\lambda^{\prime }}_{i}$ for all *i*, then

$$WNPHAM^{\left(x\right)}\left({\tilde{n}}_{1},{\tilde{n}}_{2},\cdots ,{\tilde{n}}_{n}\right)\geq WNPHAM^{\left(x\right)}\left({\tilde{n}}^{\prime }{}_{1},{\tilde{n}}^{\prime }{}_{2},\cdots ,{\tilde{n}}^{\prime }{}_{n}\right).$$ (24)

Proof. (1) Since $\mu_{i}\geq {\mu^{\prime }}_{i}$ for all *i*, then

$$\mu_{i_{j}}\geq {\mu^{\prime }}_{i_{j}},1-\gamma_{i_{j}}\leq 1-{\gamma^{\prime }}_{i_{j}}$$

(2) Since $\rho_{i}\leq {\rho^{\prime }}_{i}$ for all *i*, then

$$\rho_{i_{j}}\leq \rho_{i_{j}}^{\prime },\;1-\delta_{i_{j}}\geq 1-\delta_{i_{j}}^{\prime }$$

(3) Since $\lambda_{i}\leq {\lambda^{\prime }}_{i}$, for all *i*, then

$$\lambda_{i_{j}}\leq \lambda_{i_{j}}^{\prime },1-\lambda_{i_{j}}\geq 1-\lambda_{i_{j}}^{\prime }$$

So, we can get

$$WNPHAM^{\left(x\right)}\left({\tilde{n}}_{1},{\tilde{n}}_{2},\cdots ,{\tilde{n}}_{n}\right)\geq WNPHAM^{\left(x\right)}\left({\tilde{n}}^{\prime }{}_{1},{\tilde{n}}^{\prime }{}_{2},\cdots ,{\tilde{n}}^{\prime }{}_{n}\right).$$

(3) Theorem 8 (Boundedness). The WNPHAM operator situates within:

$$min\left({\tilde{n}}_{1},{\tilde{n}}_{2},\cdots ,{\tilde{n}}_{n}\right)\leq WNPHAM\left({\tilde{n}}_{1},{\tilde{n}}_{2},\cdots ,{\tilde{n}}_{n}\right)\leq max\left({\tilde{n}}_{1},{\tilde{n}}_{2},\cdots ,{\tilde{n}}_{n}\right)$$ (25)

Proof. Let $m=min\left({\tilde{n}}_{1},{\tilde{n}}_{2},\cdots ,{\tilde{n}}_{n}\right),M=max\left({\tilde{n}}_{1},{\tilde{n}}_{2},\cdots ,{\tilde{n}}_{n}\right)$, since $m\leq {\tilde{n}}_{i_{j}}\leq M$, according to Theorem 3, we could obtain:

$$m=min\left({\tilde{n}}_{1},{\tilde{n}}_{2},\cdots ,{\tilde{n}}_{n}\right)\leq WNPHAM\left({\tilde{n}}_{1},{\tilde{n}}_{2},\cdots ,{\tilde{n}}_{n}\right)$$

And

$$WNPHAM\left({\tilde{n}}_{1},{\tilde{n}}_{2},\cdots ,{\tilde{n}}_{n}\right)\leq max\left({\tilde{n}}_{1},{\tilde{n}}_{2},\cdots ,{\tilde{n}}_{n}\right)=M$$

Then

$$m=min\left({\tilde{n}}_{1},{\tilde{n}}_{2},\cdots ,{\tilde{n}}_{n}\right)$$

$$\leq WNPHAM\left({\tilde{n}}_{1},{\tilde{n}}_{2},\cdots ,{\tilde{n}}_{n}\right)$$

$$\leq max\left({\tilde{n}}_{1},{\tilde{n}}_{2},\cdots ,{\tilde{n}}_{n}\right)$$

### WMNPWPPHAM operator

Definition 13. Let $\eta =\left({\tilde{n}}_{1},{\tilde{n}}_{2},\cdots ,{\tilde{n}}_{n}\right)$ be a collection of MNS, and the elements could well be separated into *l* parts *P* = (*P*_1_, *P*_2_,⋯,*P*_*l*_), where $P_{t}=\left\{{\tilde{n}}_{t1},{\tilde{n}}_{t2},\cdots ,{\tilde{n}}_{t_{\left|P_{t}\right|}}\right\}$, *t* = (1, 2,⋯,*l*), *P*_*s*_ ⋂ *P*_*t*_ = ∅, $\cup_{t=1}^{l}P_{t}=\tilde{n}$, the WMNPPHAM operator is described as follows:

$$\begin{array}{l} WMNPPHAM^{p}\left({\tilde{n}}_{1},{\tilde{n}}_{2},\cdots ,{\tilde{n}}_{n}\right)\\ =\frac{1}{l}\overset{l}{\underset{t=1}{⊕}}\left(\underset{1\leq i_{1}<\cdots <i_{x}\leq \text{q}}{⊕}{\left(\overset{p}{\underset{j=1}{⊗}}n\tau_{i_{j}}{\tilde{n}}_{i_{j}}\right)}^{\frac{1}{p}}/C_{q}^{p}\right) \end{array}$$ (26)

Furthermore, the foregoing operators satisfy the Theorems of Idempotency, Commutativity, and Boundedness. However, the proofs are much like the proofs of the Theorems for MNPPHAM and WMNPPHAM operators, therefore the proof procedure is omitted here.

## MAGDM approach with WMNPPHAM operators

In this part, we will utilize the WMNPPHAM operators to solve the MAGDM issue. For example, a MAGDM issue concludes a set of m alternatives *A* = {*A*_1_, *A*_2_,…,*A*_*m*_}, the decision makers *D* = {*D*_1_, *D*_2_,…,*D*_*z*_} with their weight vector *θ* = (*θ*_1_, *θ*_2_,…,*θ*_*z*_)^*T*^ and a collection of n attributes *C* = {*C*_1_, *C*_2_,…,*C*_*n*_} with *ω* = (*ω*_1_, *ω*_2_,…,*ω*_*n*_)^*T*^ matching *θ*_*l*_ ∈ [0,1],$\sum_{l=1}^{z}\theta_{l}=1$, *ω*_*n*_ ∈ [0,1],$\sum_{j=1}^{n}\omega_{j}=1$. Taking into account the relationship of the attribute set *C*, *C* can be divided into *l* parts *P*_1_, *P*_2_,…,*P*_*l*_, where $P_{t}=\left\{C_{t1},C_{t2},\cdots ,C_{t_{\left|P_{t}\right|}}\right\}$, *t* = (1, 2,…,*l*), *P*_*s*_ ⋂ *P*_*t*_ = ∅,$\cup_{t=1}^{l}P_{t}=C$. |*P*_*t*_| represents the number in partition *P*_*t*_. Assume that the DM *D*_*h*_(*h* = 1,2,…,*z*) evaluates his/her assessment using MNS ${\tilde{n}}_{ij}{}^{h}=\left({\tilde{t}}_{ij}{}^{h},{\tilde{i}}_{ij}{}^{h},{\tilde{f}}_{ij}{}^{h}\right)$, which is the assessment of the attribute *C*_*j*_ regarding the alternative *A*_*i*_. The decision matrix might indeed be compiled as follows:

$$R^{h}={\left[{\tilde{n}}_{ij}\right]}_{m\times n}^{h}=\left[\begin{array}{cccc} {\tilde{n}}_{11}{}^{h} & {\tilde{n}}_{12}{}^{h} & \cdots & {\tilde{n}}_{1n}{}^{h}\\ {\tilde{n}}_{21}{}^{h} & {\tilde{n}}_{22}{}^{h} & \cdots & {\tilde{n}}_{2n}{}^{h}\\ \vdots & \vdots & \ddots & \vdots \\ {\tilde{n}}_{m1}{}^{h} & {\tilde{n}}_{m2}{}^{h} & \cdots & {\tilde{n}}_{mn}{}^{h} \end{array}\right]$$ (27)

To overcome this problem, the following phases of the new MAGDM technique might be taken:

Step 1. The gathered choice matrices *R*^*h*^ must be normalized into standard matrices *SR*^*h*^ by converting the cost-type to the benefit-type.


$$R^{h}={\tilde{n}}_{ij}{}^{h}=\left\{\begin{array}{l} {\tilde{n}}_{ij}=\left({\tilde{u}}_{ij}{}^{h},{\tilde{d}}_{ij}{}^{h},{\tilde{l}}_{ij}{}^{h}\right)\\ {\left({\tilde{n}}_{ij}\right)}^{c}=\left[\left({\tilde{u}}_{ij}{}^{h}\right),\left(1-{\tilde{d}}_{ij}{}^{h}\right),\left({\tilde{l}}_{ij}{}^{h}\right)\right] \end{array}\right.$$ (28)

Step 2. Calculate the supports $Sup\left({\tilde{n}}_{ij}^{k},{\tilde{n}}_{ij}^{t}\right).$


$$Sup\left({\tilde{n}}_{i}{{}_{j}}^{k},{\tilde{n}}_{i}{{}_{j}}^{t}\right)=1-d\left({\tilde{n}}_{i}{{}_{j}}^{k},{\tilde{n}}_{i}{{}_{j}}^{t}\right),i=1,2,\ldots ,n;j=1,2,\ldots ,m;k,t=1,2,..,l,k\neq t.$$ (29)

As Definition 7, $d\left({\tilde{n}}_{ij}^{k},{\tilde{n}}_{ij}^{t}\right)$ is the Hamming-Hausdorff distance.

Step 3. Estimate the weights $\sigma_{ij}^{k}$ associated with the MN ${\tilde{n}}_{ij}^{k}$.

Since

$$T\left({\tilde{n}}_{i}{{}_{j}}^{k}\right)=\sum_{t=1;t\neq k}^{l}\omega_{t}\times Sup\left({\tilde{n}}_{i}{{}_{j}}^{k},{\tilde{n}}_{i}{{}_{j}}^{t}\right);\left(k=1,2,\ldots ,l\right)$$

then the weights $\sigma_{ij}^{k}$ combined by the ${\tilde{n}}_{ij}^{k}$ could be collected:

$$\sigma_{ij}^{k}=\frac{\omega_{k}\left(1+T\left({\tilde{n}}_{ij}^{k}\right)\right)}{\sum_{k=1}^{l}\left(1+T\left({\tilde{n}}_{ij}^{k}\right)\right)};k=1,2,\ldots ,l.$$

Step 4. Aggregate each expert’s evaluation information.

To composite the MNS ${\tilde{n}}_{ij}^{k}$, use the WMNPHAM operator described in Eq (30)

n˜ij=WMNPHAMωn˜ij1,n˜ij2,…,n˜ijm=1−∏1≤i1<⋯<ix≤n1−∏j=1x∪μij∈uij1−1−μijnσij1x1Cnx,∏1≤i1<⋯<ix≤n1−∏j=1x∪ρij∈dij1−ρijnσij1x1Cnx,∏1≤i1<⋯<ix≤n1−∏j=1x∪λij∈lij1−λijnσij1x1Cnx (30)

Step 5. Estimate the supports $Sup\left({\tilde{n}}_{ij},{\tilde{n}}_{ip}\right).$


$$Sup\left({\tilde{n}}_{i}{}_{j},{\tilde{n}}_{i}{}_{p}\right)=1-d\left({\tilde{n}}_{i}{}_{j},{\tilde{n}}_{i}{}_{p}\right).$$ (31)

Step 6. Estimate the weights *τ*_*ij*_

Since

$$T\left({\tilde{n}}_{i}{}_{j}\right)=\sum_{p=1;p\neq j}^{m}w_{p}Sup\left({\tilde{n}}_{i}{}_{j},{\tilde{n}}_{i}{}_{p}\right)\left(p=1,2,\ldots ,m\right),$$

the weights *τ*_*ij*_ with the MN ${\tilde{n}}_{ij}$ can show as:

$$\tau_{i}{}_{j}=\frac{w_{j}\left(1+T\left({\tilde{n}}_{i}{}_{j}\right)\right)}{\sum_{j=1}^{m}w_{j}\left(1+T\left({\tilde{n}}_{i}{}_{j}\right)\right)}$$ (32)

Step 7. Evaluate each alternative inside each partition *P*_*t*_, using the WMNPHAM operator described by (27), where ${\tilde{n}}_{ij}=WMNPPHAM^{P_{t}}\left({\tilde{n}}^{1}{}_{i},{\tilde{n}}^{2}{}_{i},\ldots ,{\tilde{n}}^{z}{}_{i}\right)$, *i* = 1, 2,…, *m* as:

WMNPPHAMPtn˜i1,n˜i2,…,n˜in=1l⊕t=1l1CPtx⊕1≤i1<⋯<ix≤Pt⊗j=1xn˜ijnτij1x=1−∏t=1l∏1≤i1<⋯<ix≤Pt1−∏j=1x∪μij∈uij1−1−μijnτij1x1CPtx1l,∏t=1l∏1≤i1<⋯<ix≤Pt1−∏j=1x∪ρij∈dij1−ρijnτij1x1CPtx1l,∏t=1l∏1≤i1<⋯<ix≤Pt1−∏j=1x∪λij∈lij1−λijnτij1x1CPtx1l (33)

Step 8. Use the Eqs. (8) and (9) to calculate the score values $G\left({\tilde{n}}_{i}\right)$ and accuracy values $B\left({\tilde{n}}_{i}\right)$.

Step 9. As shown by Definition 6, order the alternatives *A*_*i*_ and pick the most accomplished one(s).

## Illustrative example

The investment firm seeks to choose the best option from the four agricultural brands, which are *A*_1_, *A*_2_, *A*_3_ and *A*_4_. Three characteristics are used to evaluate the four alternatives: (1) *C*_1_(the risk index), (2) *C*_2_(the growth index), (3) *C*_3_(environmental impact index), where *C*_1_ and *C*_2_ belong to the benefit type, *C*_3_ is of the cost type with their weight is *ω* = (0.35, 0.25, 0.4)^*T*^. Furthermore, there are three decision makers that can make quick choices based on their knowledge and experience. Their decisions may be written down in the form MNS by ${\tilde{n}}_{ij}^{h}=\left\{{\tilde{t}}_{n_{ij}^{h}},{\tilde{i}}_{n_{ij}^{h}},{\tilde{f}}_{n_{ij}^{h}}\right\}$ (*i* = 1,2,3,4, *j* = 1,2,3,4, *h* = 1,2,3) with their weight is *θ* = (0.3,0.5.0.2)^*T*^. The qualities may be separated into two portions based on their characteristics P1 = {C1, C2} and P2 = {C3}. In this situation, the decision maker can provide numerous possible values under each characteristic for the evaluations while adhering to the primary level of “excellent.” Then there is the decision matrix among the decision makers $R^{h}={\left[{\tilde{n}}_{ij}\right]}_{m\times n}^{h}$ could be collect as follows

$$\begin{array}{l} R^{1}=\left(\begin{array}{ccc} \langle \left\{0.4\right\},\left\{0.1\right\},\left\{0.2\right\}\rangle & \langle \left\{0.5\right\},\left\{0.2\right\},\left\{0.1\right\}\rangle & \langle \left\{0.3\right\},\left\{0.1,0.2\right\},\left\{0.4\right\}\rangle \\ \langle \left\{0.7\right\},\left\{0.1,0.2\right\},\left\{0.2\right\}\rangle & \langle \left\{0.6\right\},\left\{0.2\right\},\left\{0.2,0.3\right\}\rangle & \langle \left\{0.4\right\},\left\{0.2\right\},\left\{0.3\right\}\rangle \\ \langle \left\{0.4,0.5\right\},\left\{0.1\right\},\left\{0.3\right\}\rangle & \langle \left\{0.5\right\},\left\{0.2\right\},\left\{0.1\right\}\rangle & \langle \left\{0.4,0.5\right\},\left\{0.2\right\},\left\{0.2\right\}\rangle \\ \langle \left\{0.6\right\},\left\{0.3\right\},\left\{0.1\right\}\rangle & \langle \left\{0.5,0.6\right\},\left\{0.3\right\},\left\{0.2\right\}\rangle & \langle \left\{0.5\right\},\left\{0.1\right\},\left\{0.2\right\}\rangle \end{array}\right);\\ R^{2}=\left(\begin{array}{ccc} \langle \left\{0.6\right\},\left\{0.1\right\},\left\{0.1,0.2\right\}\rangle & \langle \left\{0.5\right\},\left\{0.2\right\},\left\{0.2\right\}\rangle & \langle \left\{0.4,0.5\right\},\left\{0.1\right\},\left\{0.3\right\}\rangle \\ \langle \left\{0.5\right\},\left\{0.2\right\},\left\{0.2\right\}\rangle & \langle \left\{0.6\right\},\left\{0.2\right\},\left\{0.1,0.2\right\}\rangle & \langle \left\{0.5\right\},\left\{0.3\right\},\left\{0.2\right\}\rangle \\ \langle \left\{0.4,0.5\right\},\left\{0.2\right\},\left\{0.1\right\}\rangle & \langle \left\{0.5\right\},\left\{0.1\right\},\left\{0.3\right\}\rangle & \langle \left\{0.5\right\},\left\{0.1\right\},\left\{0.2,0.3\right\}\rangle \\ \langle \left\{0.5\right\},\left\{0.3\right\},\left\{0.2\right\}\rangle & \langle \left\{0.8\right\},\left\{0.2,0.3\right\},\left\{0.2\right\}\rangle & \langle \left\{0.5\right\},\left\{0.2\right\},\left\{0.2\right\}\rangle \end{array}\right);\\ R^{3}=\left(\begin{array}{ccc} \langle \left\{0.4,0.5\right\},\left\{0.2\right\},\left\{0.3\right\}\rangle & \langle \left\{0.4\right\},\left\{0.2,0.3\right\},\left\{0.3\right\}\rangle & \langle \left\{0.2\right\},\left\{0.2\right\},\left\{0.5\right\}\rangle \\ \langle \left\{0.6\right\},\left\{0.1,0.2\right\},\left\{0.2\right\}\rangle & \langle \left\{0.6\right\},\left\{0.1\right\},\left\{0.2\right\}\rangle & \langle \left\{0.5\right\},\left\{0.2\right\},\left\{0.1,0.2\right\}\rangle \\ \langle \left\{0.3,0.4\right\},\left\{0.2\right\},\left\{0.3\right\}\rangle & \langle \left\{0.5\right\},\left\{0.2\right\},\left\{0.3\right\}\rangle & \langle \left\{0.5\right\},\left\{0.2,0.3\right\},\left\{0.2\right\}\rangle \\ \langle \left\{0.7\right\},\left\{0.1,0.2\right\},\left\{0.1\right\}\rangle & \langle \left\{0.6\right\},\left\{0.1\right\},\left\{0.2\right\}\rangle & \langle \left\{0.4\right\},\left\{0.3\right\},\left\{0.2\right\}\rangle \end{array}\right). \end{array}$$

### Decision-making procedure

Step 1. Normalize the decision matrix.

The normalized MN decision matrix ${\tilde{R}}^{k}={\left({\tilde{n}}_{ij}^{k}\right)}_{4\times 3}$ can be calculated using Eq (28) as:

$$\begin{array}{l} R^{1}=\left(\begin{array}{ccc} \langle \left\{0.4\right\},\left\{0.1\right\},\left\{0.2\right\}\rangle & \langle \left\{0.5\right\},\left\{0.2\right\},\left\{0.1\right\}\rangle & \langle \left\{0.4\right\},\left\{0.8,0.9\right\},\left\{0.3\right\}\rangle \\ \langle \left\{0.7\right\},\left\{0.1,0.2\right\},\left\{0.2\right\}\rangle & \langle \left\{0.6\right\},\left\{0.2\right\},\left\{0.2,0.3\right\}\rangle & \langle \left\{0.3\right\},\left\{0.8\right\},\left\{0.4\right\}\rangle \\ \langle \left\{0.4,0.5\right\},\left\{0.1\right\},\left\{0.3\right\}\rangle & \langle \left\{0.5\right\},\left\{0.2\right\},\left\{0.1\right\}\rangle & \langle \left\{0.2\right\},\left\{0.8\right\},\left\{0.4,0.5\right\}\rangle \\ \langle \left\{0.6\right\},\left\{0.3\right\},\left\{0.1\right\}\rangle & \langle \left\{0.5,0.6\right\},\left\{0.3\right\},\left\{0.2\right\}\rangle & \langle \left\{0.2\right\},\left\{0.9\right\},\left\{0.5\right\}\rangle \end{array}\right);\\ R^{2}=\left(\begin{array}{ccc} \langle \left\{0.6\right\},\left\{0.1\right\},\left\{0.1,0.2\right\}\rangle & \langle \left\{0.5\right\},\left\{0.2\right\},\left\{0.2\right\}\rangle & \langle \left\{0.3\right\},\left\{0.9\right\},\left\{0.4,0.5\right\}\rangle \\ \langle \left\{0.5\right\},\left\{0.2\right\},\left\{0.2\right\}\rangle & \langle \left\{0.6\right\},\left\{0.2\right\},\left\{0.1,0.2\right\}\rangle & \langle \left\{0.2\right\},\left\{0.7\right\},\left\{0.5\right\}\rangle \\ \langle \left\{0.4,0.5\right\},\left\{0.2\right\},\left\{0.1\right\}\rangle & \langle \left\{0.5\right\},\left\{0.1\right\},\left\{0.3\right\}\rangle & \langle \left\{0.2,0.3\right\},\left\{0.9\right\},\left\{0.5\right\}\rangle \\ \langle \left\{0.5\right\},\left\{0.3\right\},\left\{0.2\right\}\rangle & \langle \left\{0.8\right\},\left\{0.2,0.3\right\},\left\{0.2\right\}\rangle & \langle \left\{0.2\right\},\left\{0.8\right\},\left\{0.5\right\}\rangle \end{array}\right);\\ R^{3}=\left(\begin{array}{ccc} \langle \left\{0.4,0.5\right\},\left\{0.2\right\},\left\{0.3\right\}\rangle & \langle \left\{0.4\right\},\left\{0.2,0.3\right\},\left\{0.3\right\}\rangle & \langle \left\{0.5\right\},\left\{0.8\right\},\left\{0.2\right\}\rangle \\ \langle \left\{0.6\right\},\left\{0.1,0.2\right\},\left\{0.2\right\}\rangle & \langle \left\{0.6\right\},\left\{0.1\right\},\left\{0.2\right\}\rangle & \langle \left\{0.1,0.2\right\},\left\{0.8\right\},\left\{0.5\right\}\rangle \\ \langle \left\{0.3,0.4\right\},\left\{0.2\right\},\left\{0.3\right\}\rangle & \langle \left\{0.5\right\},\left\{0.2\right\},\left\{0.3\right\}\rangle & \langle \left\{0.2\right\},\left\{0.7,0.8\right\},\left\{0.5\right\}\rangle \\ \langle \left\{0.7\right\},\left\{0.1,0.2\right\},\left\{0.1\right\}\rangle & \langle \left\{0.6\right\},\left\{0.1\right\},\left\{0.2\right\}\rangle & \langle \left\{0.2\right\},\left\{0.7\right\},\left\{0.4\right\}\rangle \end{array}\right). \end{array}$$

Step 2. Compute the supports degree $Sup\left({\tilde{n}}_{ij}^{k},{\tilde{n}}_{ij}^{t}\right).$ For convenience, ${\left(Sup\left({\tilde{n}}_{ij}^{k},{\tilde{n}}_{ij}^{t}\right)\right)}_{4\times 3}$ can denoted by *Sup*^*kt*^. According to Eq (29), the *Sup*^*kt*^ (*k*,*t* = 1,2,3; *k* ≠ *t*) can be calculated:

$$\begin{array}{l} Sup^{12}=Sup^{21}=\left(\begin{array}{ccc} 0.9167 & 0.9667 & 0.9000\\ 0.9167 & 0.9667 & 0.9000\\ 0.9000 & 0.9000 & 0.9333\\ 0.9333 & 0.9000 & 0.9667 \end{array}\right),\\ Sup^{13}=Sup^{31}=\left(\begin{array}{ccc} 0.9167 & 0.8833 & 0.9167\\ 0.9667 & 0.9667 & 0.9167\\ 0.9333 & 0.9333 & 0.9667\\ 0.9167 & 0.9167 & 0.9000 \end{array}\right),\\ Sup^{23}=Sup^{32}=\left(\begin{array}{ccc} 0.8667 & 0.9167 & 0.8167\\ 0.9500 & 0.9500 & 0.9667\\ 0.9000 & 0.9667 & 0.9333\\ 0.8500 & 0.8833 & 0.9333 \end{array}\right). \end{array}$$

Step 3. Estimate the weights $\sigma_{ij}^{k}$ with ${\tilde{n}}_{ij}^{k}$.

The ${\left(T\left({\tilde{n}}_{ij}^{k}\right)\right)}_{4\times 3}$ can be calculated as *T*^*k*^ (*k* = 1,2,3):

$$\begin{array}{l} T^{1}=\left(\begin{array}{ccc} 0.6417 & 0.6600 & 0.6333\\ 0.6517 & 0.6734 & 0.6333\\ 0.6367 & 0.6367 & 0.6600\\ 0.6500 & 0.6333 & 0.6634 \end{array}\right),\\ T^{2}=\left(\begin{array}{ccc} 0.4484 & 0.4734 & 0.6784\\ 0.7500 & 0.4800 & 0.4633\\ 0.4500 & 0.4633 & 0.4677\\ 0.4500 & 0.4467 & 0.4767 \end{array}\right),\\ T^{3}=\left(\begin{array}{ccc} 0.7084 & 0.7233 & 0.6834\\ 0.7650 & 0.7600 & 0.7584\\ 0.7300 & 0.7633 & 0.7567\\ 0.7000 & 0.7167 & 0.7367 \end{array}\right). \end{array}$$

The weights $\sigma_{ij}^{k}\left(i,j=1,2,3,4;k=1,2,\ldots ,l\right)$ can be formed by MN ${\tilde{n}}_{ij}^{k}$ using Eq (31). ${\left(\sigma_{ij}^{k}\right)}_{4\times 3}$ are formed by *σ*^*k*^ (*k* = 1,2,3) as follows:

$$\begin{array}{l} \sigma^{1}=\left(\begin{array}{ccc} 0.3160 & 0.3153 & 0.2941\\ 0.2875 & 0.3149 & 0.3114\\ 0.3143 & 0.3117 & 0.3147\\ 0.3173 & 0.3148 & 0.3149 \end{array}\right),\\ \sigma^{2}=\left(\begin{array}{ccc} 0.4647 & 0.4665 & 0.5038\\ 0.5077 & 0.4642 & 0.4650\\ 0.4641 & 0.4644 & 0.4634\\ 0.4647 & 0.4647 & 0.4659 \end{array}\right),\\ \sigma^{3}=\left(\begin{array}{ccc} 0.2193 & 0.2182 & 0.2021\\ 0.2048 & 0.2208 & 0.2235\\ 0.2215 & 0.2239 & 0.2220\\ 0.2179 & 0.2206 & 0.2192 \end{array}\right), \end{array}$$

Step 4. Combine the evaluation information of every expert.

The collective multivalued neutrosophic decision matrix $\tilde{R}={\left({\tilde{n}}_{ij}\right)}_{n\times m}$ can be computed as:

$$\tilde{R}=\left(\begin{array}{ccc} \langle \left\{0.4426,0.4759\right\},\left\{0.1566\right\},\left\{0.2333,0.2572\right\}\rangle & \langle \left\{0.4507\right\},\left\{0.2230,0.2577\right\},\left\{0.2157\right\}\rangle & \langle \left\{0.3734\right\},\left\{0.8502,0.8810\right\},\left\{0.3249,0.3578\right\}\rangle \\ \langle \left\{0.5727\right\},\left\{0.1535,0.1928,0.1924,0.2355\right\},\left\{0.2355\right\}\rangle & \langle \left\{0.5810\right\},\left\{0.1804\right\},\left\{0.1990,0.2221,0.2366,0.2583\right\}\rangle & \langle \left\{0.1858,0.2231\right\},\left\{0.7765\right\},\left\{0.4830\right\}\rangle \\ \langle \left\{0.3544,0.3868,0.3831,0.4168,0.3857,0.4189,0.4171,0.4512\right\},\left\{0.1829\right\},\left\{0.2737\right\}\rangle & \langle \left\{0.4841\right\},\left\{0.1987\right\},\left\{0.2466\right\}\rangle & \langle \left\{0.1934,0.2236\right\},\left\{0.8228,0.8466\right\},\left\{0.4829,0.5160\right\}\rangle \\ \langle \left\{0.5818\right\},\left\{0.2420,0.2852\right\},\left\{0.1450\right\}\rangle & \langle \left\{0.5948,0.6328\right\},\left\{0.2140,0.2415\right\},\left\{0.2222\right\}\rangle & \langle \left\{0.1931\right\},\left\{0.8200\right\},\left\{0.4847\right\}\rangle \end{array}\right)$$

Step 5. Retrieve the supports $Sup\left({\tilde{n}}_{ij},{\tilde{n}}_{ip}\right).$

$Sup\left({\tilde{n}}_{ij},{\tilde{n}}_{ip}\right)\left(i=1,2,\ldots ,n;j,p=1,2,\ldots ,m;j\neq p\right)$ could be retrieved using Eq (31) as follows:

$$\begin{array}{l} Sup\left({\tilde{n}}_{11},{\tilde{n}}_{12}\right)=Sup\left({\tilde{n}}_{12},{\tilde{n}}_{11}\right)=0.9567;\\ Sup\left({\tilde{n}}_{11},{\tilde{n}}_{13}\right)=Sup\left({\tilde{n}}_{13},{\tilde{n}}_{11}\right)=0.7030;\\ Sup\left({\tilde{n}}_{12},{\tilde{n}}_{13}\right)=Sup\left({\tilde{n}}_{13},{\tilde{n}}_{12}\right)=0.7239;\\ Sup\left({\tilde{n}}_{21},{\tilde{n}}_{22}\right)=Sup\left({\tilde{n}}_{22},{\tilde{n}}_{21}\right)=0.9737;\\ Sup\left({\tilde{n}}_{21},{\tilde{n}}_{23}\right)=Sup\left({\tilde{n}}_{23},{\tilde{n}}_{21}\right)=0.5106;\\ Sup\left({\tilde{n}}_{22},{\tilde{n}}_{23}\right)=Sup\left({\tilde{n}}_{23},{\tilde{n}}_{22}\right)=0.5330;\\ Sup\left({\tilde{n}}_{31},{\tilde{n}}_{32}\right)=Sup\left({\tilde{n}}_{32},{\tilde{n}}_{31}\right)=0.9705;\\ Sup\left({\tilde{n}}_{31},{\tilde{n}}_{33}\right)=Sup\left({\tilde{n}}_{33},{\tilde{n}}_{31}\right)=0.6427;\\ Sup\left({\tilde{n}}_{32},{\tilde{n}}_{33}\right)=Sup\left({\tilde{n}}_{33},{\tilde{n}}_{32}\right)=0.6112;\\ Sup\left({\tilde{n}}_{41},{\tilde{n}}_{42}\right)=Sup\left({\tilde{n}}_{42},{\tilde{n}}_{41}\right)=0.9517;\\ Sup\left({\tilde{n}}_{41},{\tilde{n}}_{43}\right)=Sup\left({\tilde{n}}_{43},{\tilde{n}}_{41}\right)=0.5717;\\ Sup\left({\tilde{n}}_{42},{\tilde{n}}_{43}\right)=Sup\left({\tilde{n}}_{43},{\tilde{n}}_{42}\right)=0.5749. \end{array}$$

Step 6. Compute the weights *τ*_*ij*_ with MNS ${\tilde{n}}_{ij}.$

The weighted support ${\left(T\left({\tilde{n}}_{ij}\right)\right)}_{4\times 3}$ of the MNS ${\tilde{n}}_{ij}$ using Eq (32), by the other MNS ${\tilde{n}}_{ip}\left(p=1,2,\ldots ,m\;\text{and}\;p\neq j\right)$ can be computed.


$${\left(T\left({\tilde{n}}_{ij}\right)\right)}_{4\times 3}=\left(\begin{array}{ccc} 0.5204 & 0.6244 & 0.4270\\ 0.4477 & 0.5540 & 0.3120\\ 0.4997 & 0.5842 & 0.3777\\ 0.4666 & 0.5631 & 0.3438 \end{array}\right).$$

So the weights *τ*_*ij*_ (*j* = 1,2,…,*m*) can be computed.


$${\left(\tau_{ij}\right)}_{4\times 3}=\left(\begin{array}{ccc} 0.3526 & 0.2691 & 0.3783\\ 0.3568 & 0.2736 & 0.3696\\ 0.3566 & 0.2691 & 0.3744\\ 0.3561 & 0.2711 & 0.3729 \end{array}\right).$$

Step 7. Evaluate the information within each partition of attributes.

This step can estimate the collective evaluation values of each alternate within *P*_*t*_ by (20), which show in Table 1.

**Table 1:** The evaluation values of each alternate within partition of attributes.

	*P*_1_	*P*_2_
*A*_1_	{{0.1347, 0.1425},{0.7146, 0.7265, 0.7318, 0.7437},{0.5236, 0.5392,0.5355, 0.5510}}	{{0.1621},{0.9405, 0.9532},{0.6536, 0.6779}}
*A*_2_	{{0.1437, 0.1548},{0.6573, 0.6770, 0.6768, 0.6968}, {0.5869, 0.5988, 0.6060, 0.6164}}	{{0.0732,0.0891},{0.9107},{0.7642}}
*A*_3_	{{0.0815,0.0880,0.0941,0.0866,0.0934,0.0928,0.0999,0.0871,0.0939,0.0932,0.1003,0.0928,0.0999,0.0992,0.1065},{0.7023, 0.7115},{0.6287, 0.6425}}	{{0.0773,0.0904},{0.9296, 0.9396},{0.7614, 0.7806}}
*A*_4_	{{0.1509,0.1607},{0.7336, 0.7459, 0.7507, 0.7621},{0.5550}}	{{0.0769},{0.9287},{0.7633}}

The value ${\tilde{n}}_{i}$ of the alternative *α*_*i*_ can be calculated by WMNPPHAM operator in Table 2:

**Table 2:** The comprehensive value of the alternative.

	*P*_1_
*A*_1_	{{0.1485,0.1523},{0.8198,0.8322,0.8296,0.8420},{0.5850,0.6046,0.5916,0.6111}}
*A*_2_	{{0.1091, 0.1225},{0.7737, 0.7852, 0.7851, 0.7966},{0.6697,0.6765,0.6805,0.6805}}
*A*_3_	{{0.0794, 0.0892, 0.0823, 0.0923, 0.0820, 0.0919, 0.0851, 0.0951,0.0822,0.0922,0.0853,0.953,0.0851,0.0952,0.0883,0.0985},{0.8080, 0.8176},{0.6916, 0.7082}}
*A*_4_	{{0.1147, 0.1198},{0.8254, 0.8323, 0.8350,0.8413},{0.6509}}

Step 8. Use the Eqs. (8) and (9) to calculate the score values.

*S*_*i*_ can be calculated by Definition 5 as:

$$S_{1}=0.5738;S_{2}=0.5508;S_{3}=0.5253;S_{4}=0.5443.$$

Step 9. Order all the alternatives.

The results in Step 4, we can get *S*_1_ > *S*_2_ >*S*_4_ >*S*_3_. So, the final rank of all the alternatives could be shown as *A*_1_ ≻ *A*_2_ ≻ *A*_4_ ≻ *A*_3_.

### Influence of the parameter on the final result

The changing value of parameter x in the MNWHAM operator can be taken to demonstrate the effects on the ranking results in Table 3.

**Table 3:** Ranking results for different parameter.

	*S*_1_	*S*_2_	*S*_3_	*S*_4_	Ordering
*x* = 1	0.5949	0.5719	0.5512	0.5575	*A*_1_ ≻ *A*_2_ ≻ *A*_4_ ≻ *A*_3_
*x* = 2	0.5738	0.5508	0.5253	0.5443	*A*_1_ ≻ *A*_2_ ≻ *A*_4_ ≻ *A*_3_
*x* = 3	0.5655	0.5445	0.5184	0.5406	*A*_1_ ≻ *A*_2_ ≻ *A*_4_ ≻ *A*_3_

### Comparison analysis

The efficacy and practicality of the suggested MAGDM technique by WMNPPHAM operators must be compared and verified, thus we perform a comparative analysis using the same illustrative case. The analysis might be made from the following aspects: techniques utilizing MNS with other operators and ways using the same operators with different discrete forms of neutrosophic numbers. Then these different ranking results could be shown as α_1_ ≻ α_4_ ≻ α_2_ ≻ α_3_ [43]. Clearly, the ideal choice is α_1_, whereas the worst alternative is α_3_.

We summarize the reasons for variances in the final rankings of all the examined approaches and the suggested methodology in Table 4. Not only can our approach consider interrelationships between any two qualities, numerous arguments, and membership and nonmembership, but it also has beneficial flexibility to represent preference and capacity to describe uncertainty. Furthermore, our technique may partition the attributes into discrete portions that include both the interdependence and the independence of the attributes. So we may infer that WMNPPHAM is more practical and efficient.

**Table 4:** The comparison results of the different methods.

Aggregation operators	Interrelationship between arguments	Interactions among membership and nonmembership	The effect of model uncertainty is greater.	Partition
Algebraic [44]	No	No	No	No
BM [45]	No	No	Yes	No
HM [46]	No	Yes	No	Yes
MSM [47]	Yes	Yes	No	No
WMNPPHAM	Yes	Yes	Yes	Yes

## Conclusion

In this study, we propose the WMNPHAM and WMNPPHAM operators, which extend the Hamy mean and Power aggregation operator to the MNS. In addition, we describe the desirable qualities, create the score function, and use it to rank the choices. After that, based on the WMNPPHAM operator, we provide detailed procedures for solving MAGDM issues using multi-valued neutrosophic information. Furthermore, we compare the efficacy and practicality of the created technique to current methods.

Therefore, for addressing complex decision-making situations, these proposed novel multivalued neutrosophic aggregation operators can aggregate fuzzy information and partitioned parameters meantime, which can be used as a practical tool to solve the MADM challenges more efficiently and effectively. In the future, further research can expand them to other aspects of MADM or MAGDM, such as clustering algorithms or consistency analysis. Meanwhile, other fuzzy sets can be combined to deal with the practical problems, such as q-rung orthopair fuzzy 2-tuple linguistic sets [48], interval-valued intuitionistic fuzzy hypersoft set [49], and so on.
